# Data on biodegradation of total petroleum hydrocarbons using co-composting of cow manure/oily drill wastes

**DOI:** 10.1016/j.dib.2016.10.008

**Published:** 2016-10-24

**Authors:** Mehdi Ahmadi, Moloud Dashtestani, Afshin Takdastan, Sahand Jorfi, Bahman Ramavandi

**Affiliations:** a Environmental Technologies Research Center, Ahvaz Jundishapur University of Medical Sciences, Ahvaz, Iran; b Department of Environmental Health Engineering, Ahvaz Jundishapur University of Medical Sciences, Ahvaz, Iran; c Department of Environmental Engineering, Ahvaz Science and Research Branch, Islamic Azad University, Ahvaz, Iran; d Department of Environmental Health Engineering, Bushehr University of Medical Sciences, Bushehr, Iran

**Keywords:** Oil based drilling wastes, Co- composting, Petroleum hydrocarbon, Bioremediation, Cow manure

## Abstract

Oil drill cuttings are challenging wastes in oil sites especially in Khuzestan province, a major oil producing region in Iran. As co- composting is a simple and eco- friendly technique for bioremediation of oil base drill cutting, this data article designed to describe co- composting of oil base drill cutting with cow manure. The data suggest that with optimized mixture of cow manure/oily drill wastes (here, 20:1) could engender more effective treatment of the wastes (with final total petroleum hydrocarbon of 0.01 g/Kg). The data will be informative for oil drilling companies and environmental agencies for choosing it as a practical bioremediation process of soil/wastes polluted by petroleum hydrocarbons.

**Specifications Table**

**Table:** 

Subject area	*Environmental Engineering*
More specific subject area	*Bioremediation*
Type of data	*Table*
How data was acquired	– *Data gathered from 4 pilots study with various cow manure/oil drilling wastes.* – *The petroleum hydrocarbon was analyzed using a gas chromatograph coupled with a flame ionization detector.*
Data format	*Analyzed*
Experimental factors	*TPH monitoring during 2 month of experiment for determination of best mixing ratio of cow manure/oil base drill cutting to bioremediation of drill cutting waste.*
Experimental features	*Bioremediation of oil base drill cutting by using co- composting process*
Data source location	*Ahvaz, Iran, 31*°*19*′*13*″*N 48*°*40*′*09*″*E*
Data accessibility	*Data are available in the article*

**Value of the data**

• This data provide a response for the question of “how oily wastes from crude oil drilling could be biologically degraded by co-composting technique as eco-friendly technique?”
• This data article focused on the greatest challenge of oil drilling companies i.e., environmental pollution by petroleum hydrocarbons, thus, this data can be use by these companies for bioremediation of their wastes.
• This data will be interesting for environmentalist with concern on soil pollution by oil as in this data set the progress in petroleum hydrocarbons bioremediation was obtained using only cow manure (and no use of any supplements).

## 1 Data

Data presented here describe the characteristics of oil base drill cutting waste and feasibility study of co- composting with cow manure. Table 1 shows characteristics of oil base drill cutting and cow manure in the beginning of the experiments. Table 2 illustrates the total petroleum hydrocarbons (TPH) concentration during the experiments in four pilots with different oil base cutting/cow manure ratio. Mineralization of organic pollutant by biological process during bioremediation of drill cutting showed in Table 3. Table 4 shows C/N ratio during the experiments. Table 5 depicts work plan conducted in the study.

## 2 Experimental design, materials and methods

### 2.1 Drill cuttings sample

The oil based drill cuttings wastes were collected from a pit beside a constructing oil well in an oil site in the Khuzestan province, Iran. The drilling fluid used in the oil well constructing was diesel fluid. Drilling waste sample was collected with a sterile scoop from up to a depth of 10–50 cm drilling fluid and put in the sterile polythene bag. The sample was transferred to the laboratory within 3 h in darkness and refrigerated condition (4 °C) for avoiding any photo-oxidation of hydrocarbons. The concentrations of TPHs, N, C, pH, and humidity were initially analyzed.

### 2.2 Cow manure

Cow manure was obtained from a village near to Ahvaz city with characterization given in Table 1.

### 2.3 Experimental design

The experiments were done in 4 pilots (with approximately volume of 2 L) with a different mixing ratio of cow manure/drill cuttings and TPH, pH, N, P, C/N, moisture, and volatile suspended solids (VSS) were monitored every 15 days during 2 month period of study. The ratio of C/N in the pilots regulated in the range of 20–30/1 at the start of the tests and humidity kept in the range of 50–60%. Table 5 shows the work plan of the study.

### 2.4 Measuring methods

The anhydrous sodium sulphate and hexane procedure with slightly modification using sulphuric acid digestion was applied for TPH extraction [1], [2]. About 3 g (treated or untreated) drill cuttings sample was mixed with 6 g of anhydrous sodium sulphate in a sealed glass. After that 8 mL hexane and 170 mL sulphuric acid was added to the mixture and vigorously agitated, as well sonicated in a water bath. After another shaking the mixture by hand, 0.1 mL of the supernatant was mixed with 0.9 mL of hexane and poured in a 2 mL glass phial. The TPH was then measured using a 6890N gas chromatograph coupled with a flame ionization detector (Agilent Technologies Inc., USA) which was previously calibrated with TPH standards. The mixture pH was analyzed in an agitated 1:5 material: deionized water suspension that was allowed to equilibrate for about 1.5 h [1], [3]. The pH measurement was done using a Hach pH meter calibrated with standardized pH buffer solutions. The humidity of the drilling waste sample was measured by gravimetric method based on EPA 1684 method [4]. The organic matter content of the samples was also determined by EPA 1684 [4].

## Figures and Tables

**Table 1:** Characteristics of oil base drill cutting and cow manure.

Characteristics	Drill Cutting	Cow manure
TPH (g/Kg)	360.46	0
N (%)	0.44	3.49
C (%)	55.19	55.44
Humidity (%)	9.25	75.66
Total solids (%)	90.75	24.34

**Table 2:** TPH concentration (g/Kg) during the experiment.

Pilot	Days of experiment				
	0	15	30	45	60
A	269.55	142.90	126.42	90.89	83.46
B	47.22	27.35	8.91	1.54	0.82
C	22.54	7.40	1.84	0.37	0.24
D	12.43	2.99	0.31	0.10	0.01

**Table 3:** Volatile solids content (%) during the experiment.

Pilot	Days of experiment				
	0	15	30	45	60
A	14.16	13.92	13.44	13.22	12.90
B	47.02	43.23	40.84	40.09	39.80
C	63.35	56.98	52.72	48.60	43.70
D	69.95	60.77	54.78	55.32	44.75

**Table 4:** C/N ratio fluctuation during the experiment.

Pilot	Days of experiment				
	0	15	30	45	60
A	100.2	108.1	131.2	134.4	137.8
B	46.5	47.6	50.2	59.4	69.8
C	24.2	25.5	30.7	46.0	57.6
D	16.5	17.2	20.5	30.4	43.5

**Table 5:** Work plan of the study.

Pilot	Cow manure to drill cutting ratio
A	0
B	5.6
C	10
D	20
